# Attitudes of healthcare workers toward recommending vaccination against herpes zoster: a study based on the health belief model and theory of planned behavior

**DOI:** 10.3389/fpubh.2026.1710056

**Published:** 2026-03-25

**Authors:** Bingjie Wang, Zhenwei Li

**Affiliations:** Zhejiang Chinese Medical University, Hangzhou, China

**Keywords:** attitude measurement, health belief model, healthcare workers, herpes zoster, theory of planned behavior, vaccine promotion

## Abstract

**Objective:**

To examine Chinese healthcare workers’ (HCWs’) attitudes toward promoting the herpes zoster (HZ) vaccine, using the Health Belief Model (HBM), the Theory of Planned Behavior (TPB), and dissemination theories.

**Methods:**

A cross-sectional survey was conducted among HCWs from multiple provinces and provincial-level municipalities in China between November 2023 and February 2024. The questionnaire assessed HZ-related knowledge, constructs derived from the Health Belief Model, subjective norms from the Theory of Planned Behavior, and dissemination- and organizational-level factors. Hierarchical multiple linear regression analyses were performed to examine associations with attitudes toward HZ vaccine promotion.

**Results:**

Healthcare workers’ demonstrated an overall moderate level of knowledge regarding HZ and its vaccination. In regression analyses, perceived severity, perceived benefits, perceived barriers, and self-efficacy were significantly associated with attitudes toward HZ vaccine promotion, whereas perceived susceptibility was not. Subjective norms, leadership involvement, and perceived timelines for vaccination implementation were also positively associated with attitudes. The explanatory power of the models increased sequentially with the inclusion of TPB and organizational factors.

**Conclusion:**

While Chinese healthcare workers generally support HZ vaccine promotion, gaps remain in policy awareness and personal vaccination uptake. Using an integrated framework informed by the HBM, TPB, and dissemination theory, this study shows that HCWs’ attitudes toward HZ vaccine promotion are shaped by both individual belief-based factors and organizational context. Interventions aiming to strengthen HZ vaccine promotion should address perceived benefits, barriers, and self-efficacy while fostering supportive institutional environments.

## Introduction

Herpes zoster (HZ), caused by reactivation of latent varicella-zoster virus (VZV), affects approximately one in three individuals during their lifetime (1–3). The incidence rises sharply after the age of 50, reflecting age-related decline in cellular immunity (4, 5). Postherpetic neuralgia (PHN), the most common complication, develops in 10–50% of older patients and can persist for months or years, causing chronic pain, reduced functional capacity, and impaired quality of life (6–8). Globally, the aging population is expected to substantially increase the burden of HZ and its complications (2, 9). Epidemiological studies from North America, Europe, and Asia consistently report HZ incidence between 3 and 10 per 1,000 person-years (3, 10, 11).

Zoster vaccine has demonstrated high efficacy and durability in randomized trials, but uptake in real-world practice is constrained by limited awareness, concerns over safety and efficacy, and cost barriers (12–14). Vaccination decisions are not made in isolation. Healthcare workers (HCWs) play a pivotal role in shaping public perceptions, influencing patient decisions, and implementing immunization policies (15, 16). Studies in influenza and COVID-19 vaccination show that HCWs’ knowledge, attitudes, and behaviors strongly predict patient uptake (17, 18). Despite this, few studies have systematically examined HCWs’ perspectives on HZ vaccination.

Theoretical frameworks provide a lens to understand vaccine-related behaviors. The Health Belief Model (HBM) emphasizes perceived susceptibility, severity, benefits, and barriers as determinants of preventive actions (19). The Theory of Planned Behavior (TPB) integrates attitudes, subjective norms, and perceived behavioral control in predicting intention and behavior (20). Dissemination theory highlights organizational context and leadership in implementing health innovations (21). Applying these frameworks together allows a comprehensive understanding of HCWs’ motivations and constraints regarding HZ vaccine promotion.

Several gaps remain. Most existing studies have focused on patients rather than recommenders, overlooking the crucial intermediary role of HCWs. Moreover, empirical evidence linking theoretical constructs (HBM, TPB, dissemination) with actual attitudes and intentions among HCWs remains scarce (22, 23).

To address these gaps, we conducted a large cross-sectional survey of Chinese HCWs across different institutions and specialties. We aimed to (1) assess their knowledge, attitudes, and willingness to promote the HZ vaccine; (2) identify perceived facilitators and barriers, using the HBM and TPB as guiding frameworks; (3) explore organizational and dissemination-related factors such as leadership involvement and workplace adoption; and (4) provide evidence-based recommendations to improve HZ vaccine coverage in China.

### Hypotheses

Guided by the Health Belief Model (HBM), the Theory of Planned Behavior (TPB), and dissemination theory, this study proposes the following hypotheses regarding healthcare workers’ (HCWs) attitudes and intentions to promote herpes zoster (HZ) vaccination.

### HBM-related hypotheses

HCWs’ perceptions of HZ and its prevention are expected to be associated with their attitudes toward promoting the HZ vaccine. Specifically, higher levels of perceived susceptibility to HZ and perceived severity of HZ outcomes are hypothesized to be positively associated with more favorable attitudes toward HZ vaccine promotion (H1a–H1b). In addition, greater perceived benefits of HZ vaccination and higher self-efficacy in vaccine-related communication are expected to be positively associated with promotion attitudes, whereas greater perceived barriers are expected to be negatively associated (H1c–H1e).

### TPB-related hypotheses

Consistent with the Theory of Planned Behavior, stronger subjective norms—reflecting perceived expectations from colleagues, institutions, and patients—and higher perceived behavioral control are expected to be positively associated with more favorable attitudes toward HZ vaccine promotion (H2).

### Dissemination-related hypotheses

Organizational and dissemination-related factors are also expected to play an important role. Stronger leadership involvement, and clearer institutional timelines for achieving vaccination targets are hypothesized to be positively associated with HCWs’ intentions to promote the HZ vaccine (H3a–H3b).

## Method

### Study design

We conducted a cross-sectional survey of HCWs from November 2023 to February 2024. Participants were recruited from multiple provinces and provincial-level municipalities across China, covering a range of healthcare institutions and clinical settings.

### Questionnaire development and measures

A structured, self-administered questionnaire was developed based on established behavioral theories and prior vaccination research. The questionnaire comprised the following sections:

#### Sociodemographic and professional characteristics

Information was collected on participants’ age, gender, profession, professional rank, educational attainment, years of service, type of healthcare institution, and average monthly patient volume.

#### Knowledge related to herpes zoster and vaccination

HZ-related knowledge was assessed using five items addressing key domains, including the causative pathogen, common complications, treatment options, preventive strategies, and current vaccination recommendations. A composite knowledge score was calculated by summing correct responses, with higher scores indicating greater knowledge.

#### Health belief model (HBM) constructs

Perceptions relevant to HZ vaccine promotion were assessed across five HBM domains: perceived susceptibility, perceived severity, perceived benefits, perceived barriers, and self-efficacy. Items were designed to capture HCWs’ beliefs about personal and patient risk, the clinical and preventive value of vaccination, and perceived obstacles to recommending the HZ vaccine in practice. Internal consistency analyses indicated acceptable to good reliability across all HBM subscales (Cronbach’s *α* = 0.57). Item-total correlations were examined to ensure that each item contributed meaningfully to its respective construct.

#### Theory of planned behavior (TPB) constructs

TPB-related variables included attitudes toward promoting the HZ vaccine, subjective norms (perceived expectations from colleagues, institutions, and patients) in routine clinical work. Cronbach’s alpha coefficients demonstrated satisfactory internal consistency for all TPB subscales (*α* = 0.69), supporting the reliability of these measures in the present sample.

#### Dissemination and organizational factors

Organizational context was assessed by leadership involvement in vaccination-related initiatives, and perceived timelines for achieving institutional vaccination targets

#### Additional influencing factors

Participants were also asked to evaluate the influence of broader contextual factors on their vaccine promotion behaviors, including professional knowledge, patient demand, government policies, social and cultural factors, and peer influence.

### Statistical analysis

All statistical analyses were conducted to examine associations between HCWs’ knowledge, and attitudes regarding HZ vaccine promotion, guided by HBM, TPB, and dissemination theory.

Descriptive statistics were first used to summarize participants’ sociodemographic and professional characteristics, as well as distributions of knowledge scores, theoretical constructs, and promotion-related attitudes and intentions. Categorical variables were presented as frequencies and percentages.

Internal consistency of multi-item scales was assessed using Cronbach’s alpha coefficients, with values ≥0.50 considered indicative of acceptable reliability.

Associations between key variables were examined using Pearson correlation analyses to explore relationships among HZ-related knowledge, HBM constructs, TPB constructs, organizational factors, and promotion attitudes and intentions.

To identify independent predictors, multiple linear regression analyses were performed. First, attitudes toward HZ vaccine promotion were modeled as the dependent variable, with HBM constructs entered as predictors. TPB constructs were added in the second step, and dissemination and organizational factors were included in the final step. Changes in explained variance (*R*^2^) were examined to assess the incremental contribution of TPB and organizational factors beyond HBM constructs alone.

Standardized regression coefficients (*β*), standard errors (SE), t statistics, and corresponding *p* values were reported to facilitate interpretation of effect sizes and statistical significance.

Significance was set at *p* < 0.05. Data were analyzed using SPSS 27.0.

## Results

### Study population

A total of 500 questionnaires were collected; 422 were valid (response rate 84.4%). Respondents included 174 men (42.2%) and 238 women (57.8%). The majority were aged 20–39 years (77.3%). Occupations included clinical physicians (41.5%), traditional Chinese medicine physicians (23.5%), and others across specialties. Only 8.5% of respondents had received the HZ vaccine themselves. Detailed sociodemographic and professional characteristics of the study population are presented in Table 1.

**Table 1:** Sociodemographic and professional characteristics of participants.

Characteristics	*N*	Percentage
Sex		
Male	168	39.8
Female	254	60.2
Age		
20-	164	38.9
30-	162	38.4
40-	63	14.9
50-	33	7.8
Position		
Clinicians	151	35.8
Traditional Chinese physician	91	21.6
Nurse	48	11.4
Public health physician	14	3.3
Other	118	28.0
Department		
Internal medicine	94	22.3
Dermatology department	10	2.4
Pain management	17	4.0.0
Department of Public Health	25	5.9
Other	276	65.4
Educational level		
Associate degree	39	9.2
Bachelor’s degree	262	62.1
Master’s degree	112	26.5
Doctor’s degree	9	2.1
Tenure/years		
<5	149	35.3
5–9	129	30.6
10–19	79	18.7
20-	65	15.4
Institution type		
Tertiary hospitals	236	55.9
Secondary hospital	76	18.0
Primary hospital	28	6.6
Other hospital	46	10.9
Preventive health care institution	36	8.5
Professional title		
Primary	162	38.4
Intermediate/Supervisory	145	34.4
Associate chief physician	42	10.0
Chief physicians	11	2.6
Other	62	14.7
Number of patients per month		
<50	180	42.7
50-	97	23.0
101-	58	13.7
201-	39	9.2
501-	48	11.4

### Knowledge of healthcare workers regarding herpes zoster and zoster vaccination

As presented in Table 2, healthcare workers (HCWs) demonstrated an overall moderate level of knowledge regarding HZ. All most participants (>95%) correctly identified the viral etiology of HZ and recognized postherpetic neuralgia as a common complication. Awareness of antiviral therapy as the main treatment was also high.

**Table 2:** Knowledge of healthcare workers regarding herpes zoster and zoster vaccination.

Variables	Frequency	Percentage
Knowledge of herpes zoster		
Good	279	66.1
Poor	143	33.9
Known caused by varicella-zoster virus		
Yes	409	96.9
No	13	3.1
Known common complications is PHN		
Yes	404	95.7
No	18	4.3
Known treatment mainly is antiviral drugs		
Yes	369	87.4
No	53	12.6
Known the most effective method is vaccination		
Yes	294	69.7
No	128	30.3
Known the recommended vaccination age		
Yes	111	26.3
No	311	73.7

With respect to prevention, the majority of HCWs acknowledged vaccination as the most effective preventive measure; however, gaps were observed in knowledge of vaccination-specific recommendations, particularly regarding the recommended age for HZ vaccination.

### Correlations between knowledge, behavioral constructs, and promotion intentions

Pearson correlation analyses were conducted to examine bivariate associations between HZ-related knowledge, HBM constructs, TPB constructs, dissemination-related factors, and promotion intentions. As shown in Table 3, attitudes of HCWs toward recommending vaccination were significantly correlated with perceived susceptibility, perceived severity, perceived benefits, perceived barriers, self-efficacy, subjective norms, leadership, and timeline.

**Table 3:** Pearson correlations between key variables.

Variables	Attitudes	Age	Sex	Work year	Score	Perceived susceptibility	Perceived severity	Perceived benefits	Self-efficacy	Perceived barriers	Subjective norms	Leadership involvement	Perceived timelines
Attitudes	1.00	0.05	0.13^**^	0.02	0.10^*^	0.17	0.24^***^	0.26^***^	0.31^***^	−0.13^**^	0.35^***^	0.29^***^	0.16^***^
Age	0.05	1.00	−0.17^***^	0.86^***^	0.14^**^	0.01	0.03	0.15	0.01	0.04	−0.02	0.04	0.05
Sex	0.13^**^	−0.17^***^	1.00	−0.19^***^	0.02	0.11	0.06	0.05	−0.05	−0.06	0.12	0	0.13
Work year	0.02	0.86^***^	−0.19^***^	1.00	0.14^**^	0	−0.01	0.15	−0.02	0.01	0.02	0.05	0.02
Score	0.10^*^	0.14^**^	0.02	0.14^**^	1.00	0.15	0.13	0.04	0.05	−0.12	0	0.11	0.1
Perceived susceptibility	0.17^***^	0.04	0.15^***^	0.03	0.12^*^	1.00^***^	0.27^***^	0.26^***^	0.17^***^	0.03	0.14^**^	0.07	0.05
Perceived severity	0.24^***^	0.01	0.11^*^	0.00	0.15^**^	0.27^***^	1.00	0.32^***^	0.21^***^	0.07	0.06	0.14^**^	0.14^**^
Perceived benefits	0.26^***^	0.03	0.06	−0.01	0.13^**^	0.26	0.32^***^	1.00	0.27^***^	−0.07	0.27^***^	0.17^***^	0.07
Self-efficacy	0.31^***^	0.15^**^	0.05	0.15^**^	0.04	0.17^***^	0.21^***^	0.27^***^	1.00	−0.10^*^	0.26^***^	0.18^***^	0.00
Perceived barriers	−0.13^**^	0.01	−0.05	−0.02	0.05	0.03	0.07	−0.07	−0.10^*^	1.00	−0.16^***^	−0.08	0.09
Subjective norms	0.35^***^	0.04	−0.06	0.01	−0.12^*^	0.14^**^	0.06	0.27^***^	0.26^***^	−0.16^***^	1.00	0.15^**^	−0.20^***^
Leadership involvement	0.29^***^	−0.02	0.12	0.02	0.00	0.07	0.14^**^	0.17^***^	0.18^***^	−0.08	0.15^**^	1.00	0.16^**^
Perceived timelines	0.16^***^	0.04	0.00	0.05	0.11	0.05	0.14^**^	0.07	0.00	0.09	−0.20^***^	0.16^**^	1.00

*P < 0.05, **p < 0.01, ***p < 0.001.

### Predictors of attitudes toward HZ vaccine promotion

As shown in Table 4, a series of hierarchical multiple linear regression models were constructed to examine predictors of HCWs intention to promote HZ vaccination.

**Table 4:** Multiple linear regression analysis of HBM, TPB and dissemination and organizational factors constructs predicting attitudes toward HZ vaccine promotion.

Variables	HBM				HBM + TPB				HBM + TPB + dissemination and organizational factors			
	*β*	SE	*t*	*p*-value	*β*	SE	*t*	*p*-value	*β*	SE	*t*	*p*-value
Multiple *R*^2^	0.16				0.22				*0.28*			
Adjusted *R*^2^	0.15				0.21				0.27			
(Intercept)	2.29	0.27	8.58	<0.001	1.45	0.30	4.82	<0.001	0.87	0.30	2.86	0.004
Perceived susceptibility	0.05	0.04	1.33	0.185	0.03	0.04	0.95	0.344	0.03	0.03	0.89	0.372
Perceived severity	0.11	0.04	2.86	0.004	0.13	0.04	3.29	0.001	0.10	0.04	2.66	0.008
Perceived benefits	0.13	0.05	2.683	0.008	0.08	0.05	1.61	0.109	0.05	0.05	1.04	0.301
Perceived barriers	−0.07	0.03	−2.46	0.014	−0.05	0.03	−1.81	0.070	−0.05	0.03	−1.89	0.060
Self-efficacy	0.11	0.02	4.76	<0.001	0.08	0.02	3.81	<0.001	0.07	0.02	3.48	<0.001
Subjective norms					0.14	0.03	5.48	<0.001	0.16	0.03	6.12	<0.001
Leadership									0.12	0.03	3.61	<0.001
Timeline									0.10	0.03	4.04	<0.001

*β* indicates standardized regression coefficients; SE, standard errors, *t* statistics.

In the model including only HBM constructs, perceived benefits and self-efficacy were positively associated with promotion intention, whereas perceived barriers showed a negative association. This model explained 16% [*R*^2^ = 16%] of the variance in promotion intention.

After adding Theory of Planned Behavior (TPB) constructs, attitudes toward HZ vaccine promotion and subjective norms emerged as significant positive predictors. The inclusion of TPB variables substantially improved model performance, with the explained variance increasing to 22% [*R*^2^ = 22%], compared with the HBM-only model.

In the fully adjusted model incorporating dissemination and organizational factors, attitudes, subjective norms, self-efficacy, and organizational factors—including leadership involvement and perceived timelines for achieving vaccination targets—remained significantly associated with promotion intention. This final model demonstrated the highest explanatory power, accounting for 28% [*R*^2^ = 28%] of the variance in promotion intention.

### Summary of hypothesis testing

A summary of hypothesis testing results is provided in Table 5. Overall, hypotheses related to individual-level behavioral constructs and organizational-level factors were partially supported.

**Table 5:** Summary of hypothesis testing.

Hypothesis	Description	Supported
H1a	Perceived susceptibility → Attitudes	No
H1b	Perceived severity → Attitudes	Yes
H1c	Perceived benefits → Attitudes	Yes
H1d	Perceived barriers → Attitudes	Yes
H1e	Self-efficacy → Attitudes	Yes
H2	Subjective norms → Attitudes	Yes
H3a	Leadership involvement → Attitudes	Yes
H3b	Timeline → Attitudes	Yes

Hypotheses were considered supported if the corresponding regression coefficients were statistically significant at *p* < 0.05.

Among Health Belief Model (HBM)–related hypotheses, perceived severity, perceived benefits, perceived barriers, and self-efficacy were significantly associated with attitudes toward herpes zoster (HZ) vaccine promotion, supporting hypotheses H1b–H1e. In contrast, perceived susceptibility was not significantly associated with attitudes, and H1a was not supported.

With respect to Theory of Planned Behavior and organizational factors, subjective norms were positively associated with attitudes toward HZ vaccine promotion, supporting H2. In addition, leadership involvement and perceived timelines for vaccination implementation were both significantly associated with attitudes, supporting hypotheses H3a and H3b, respectively.

## Discussion

This study provides a comprehensive analysis of Chinese HCWs’ perspectives on HZ vaccine promotion through the lenses of HBM, TPB, and dissemination theory. Several key insights emerge. Overall, the findings indicate that attitudes toward HZ vaccine promotion are shaped by a combination of individual belief-based factors and organizational–contextual influences, supporting the value of a multidimensional behavioral framework (22, 24). Most hypothesized associations were supported, with the exception of perceived susceptibility, highlighting the relative importance of perceived severity, benefits, self-efficacy, and contextual factors in shaping HCWs’ attitudes.

The HCWs showed generally favorable attitudes but low vaccination uptake (8.5%), reflecting a gap between theoretical support and personal practice. Second, safety and efficacy concerns dominated, echoing public hesitancy (15, 23). Third, education, cost reduction, and institutional support were viewed as the most effective strategies to promote uptake. Finally, professional rank and personal vaccination history were strongly associated with intention and knowledge, underscoring the role of experience and authority in shaping promotional behaviors.

### HBM-related findings and interpretation

Within the HBM, perceived severity, perceived benefits, and self-efficacy were positively associated with healthcare workers’ attitudes toward HZ vaccine promotion, whereas perceived barriers were negatively associated with these attitudes. These findings are in line with prior applications of the HBM, which emphasize that perceived disease consequences, expected benefits, and perceived obstacles are central determinants of preventive health behaviors (22).

Notably, perceived susceptibility was not significantly associated with attitudes. This suggests that HCWs’ attitudes toward vaccine promotion may be less influenced by their own perceived risk and more by professional judgment regarding disease impact and intervention value. Similar patterns have been observed in studies of HCWs and other professional populations, where personal risk perception plays a limited role compared with professional norms and responsibilities (25, 26).

The positive association between self-efficacy and attitudes further underscores the importance of confidence in vaccine-related communication. Previous research has shown that higher self-efficacy among HCWs is associated with greater engagement in vaccine recommendation and counseling behaviors (15, 27).

In accordance with the Theory of Planned Behavior, subjective norms were positively associated with attitudes toward HZ vaccine promotion. This finding is consistent with TPB-based research demonstrating that perceived social expectations and professional norms exert a strong influence on intention formation and attitudinal orientation in health-related behaviors (22, 28). Beyond individual-level TPB constructs, organizational and dissemination-related factors—specifically leadership involvement and perceived timelines for vaccination implementation—were also positively associated with attitudes. These results align with dissemination and implementation research indicating that leadership engagement and organizational readiness are critical for the uptake and normalization of evidence-based practices in healthcare settings (29, 30).

Taken together, these findings suggest that HCWs’ attitudes toward vaccine promotion are embedded within broader social and organizational contexts rather than being driven solely by individual beliefs. Institutional strategies that actively engage leadership and professional opinion leaders may be particularly effective in normalizing vaccine promotion and reducing attitudinal–behavioral gaps (30, 31).

The findings have important implications for vaccination practice and policy. Interventions aimed at strengthening HCWs’ engagement in HZ vaccine promotion should focus on enhancing perceived benefits, reducing perceived barriers, and improving self-efficacy through targeted education and communication training. At the organizational level, visible leadership endorsement, clear institutional guidance, and defined implementation timelines may help foster supportive normative environments for vaccine promotion.

These results indicate that strategies focusing exclusively on individual knowledge or risk perception may be insufficient without concurrent organizational support.

This study benefits from the application of an integrated theoretical framework and the inclusion of organizational-level determinants alongside individual behavioral factors.

In mainland China, an expert consensus for adults with autoimmune rheumatic diseases (2023 edition) includes HZ vaccination among recommended adult immunizations, offering operational guidance for vaccinators and rheumatology clinicians (32). The Italian SIDeMaST guidance for dermatologic diseases highlights individualized risk–benefit assessment and clinician counseling (33), while the Associazione Italiana di Oncologia Medica (AIOM) position paper recommends vaccination strategies for patients with solid tumors and emphasizes integration into routine cancer care (34). Collectively, these documents suggest that improving HZ vaccine uptake is a global, cross-specialty priority and further supports strengthening healthcare workers’ capacity and organizational support to promote vaccination.

Nevertheless, several limitations should be acknowledged. First, the cross-sectional design precludes causal inference, and the observed associations should be interpreted as correlational. Second, although internal consistency was acceptable, the questionnaire relied on adapted rather than fully validated measures specific to HZ vaccine promotion. Third, the study assessed attitudes rather than observed recommendation behaviors, which may not fully capture actual practice. Finally, the findings reflect the healthcare and policy context in China and may have limited generalizability to other settings.

Future research could employ longitudinal designs to examine changes in HCWs’ attitudes over time and to assess whether favorable attitudes translate into sustained vaccine promotion behaviors. Qualitative research may provide deeper insight into the limited role of perceived susceptibility and the mechanisms through which organizational signals influence professional norms. Further studies should also explore whether HCWs’ attitudes are associated with downstream patient vaccination uptake.

## Conclusion

In summary, this study demonstrates that healthcare workers’ attitudes toward promoting HZ vaccination are associated with perceived severity, benefits, barriers, self-efficacy, and organizational-contextual factors, but not with perceived susceptibility. By integrating HBM, TPB, and dissemination perspectives, the findings provide a nuanced understanding of the determinants of vaccine promotion attitudes and underscore the importance of addressing both individual beliefs and organizational environments to strengthen HZ vaccine promotion.

## Data Availability

The raw data supporting the conclusions of this article will be made available from the corresponding author upon reasonable request, subject to institutional approvals.
